# An examination of home-based end-of-life care for cancer patients: a qualitative study

**DOI:** 10.1186/s12904-019-0501-y

**Published:** 2019-12-16

**Authors:** Xiao Bin Lai, Li Qun Chen, Shu Hui Chen, Hai Ou Xia

**Affiliations:** School of Nursing, Fudan University, 305 Fenglin Road, Shanghai, 200032 China

**Keywords:** End-of-life care, Home care, Qualitative research, Ontology care

## Abstract

**Background:**

Only a small number of patients have utilized the home-based end-of-life care service in Shanghai that has been offered since 2012. This study explores how home-based end-of-life care is delivered in community health service centers in Shanghai and examines the difficulties in the delivery of the care.

**Methods:**

This was a qualitative study in which data were collected from interviews and analyzed using qualitative content analysis. Nineteen health care providers with experience in delivering home-based end-of-life care in 12 community health service centers were recruited. The interviews were conducted between August 2018 and February 2019.

**Results:**

Four themes emerged from the interviews: (i) Patients under home-based end-of-life care: Patients receiving the care were cancer patients with less than 1 year of life expectancy. The criteria for patients were broad. (ii) Service structure: The service was delivered regularly by the physicians and nurses using the approaches of home visits and/or telephone follow-ups. (iii) Service process: The service consisted of multiple components, including monitoring the patient’s condition, managing the patient’s symptoms, giving daily care instructions, performing nursing procedures, and giving psychological support. However, most of the care focused on monitoring the patients and managing their physical discomfort. (iv) Difficulties in delivering care: Being unable to provide the service and feeling powerless when facing psycho-spiritual problems were the two major difficulties. Three factors contributed to the suspension of the service: The gap between the service and the needs of the patients, a lack of patients, and low work motivation. The demand that the truth be concealed from the families and their attitude of avoiding talking about death were the key factors of the failure of psycho-spiritual care.

**Conclusions:**

Several issues should be addressed before the service can be further developed, including fully understanding the needs and preferences of local patients and their families, securing more financial support and a better supply of drugs, delivering better training for staff, and ensuring greater rewards for individuals and institutions providing the service.

## Background

Spending the last months at home until death is the wish of many patients and their families. Previous studies conducted in different areas have supported this observation [1–7]. In a study conducted in three metropolitan cities, more than half of the elderly surveyed (56%) in New York, Dublin, and London reported that they preferred to die at home [2]. Patients with cancer (47%) in an Australian study also wanted to be cared for at home at the end-of-life stage [3]. More than half of elderly people (64%) in an Indian study likewise wished to die at home [4]. In a study conducted in Singapore with a small sample, most cancer patients (89%) and their families also preferred home as the place of care at the end-of-life stage [5]. In China, a study found that 43.2% of elderly people with a single child in Shanghai preferred to be cared for at home at this stage [6]. In a population-based survey conducted in Hong Kong, 31.2% of the respondents preferred to die at home [7].

To realize this wish, home-based end-of-life services have been developed, especially in developed countries. The benefits of home-based end-of-life care have been demonstrated in previous studies. It can reduce the number of emergency room visits [8], admissions to intensive care units [8], hospitalizations [9], hospital deaths, and health care costs [10]. It can also increase the length of the home stays of patients [11] and the rate of home deaths [8, 12, 13]. Those patients who received home-based palliative care and their families also felt safe and hopeful [14]. Despite many studies of home-based end-of-life care, further investigation is required of the impact of the service on patients and caregivers [13].

In keeping with the international trend of developing home-based care for patients at the end-of-life stage, a home-based end-of-life care program was established in Shanghai in 2012 as part of the city’s community-based end-of-life care system, yet thus far only a few studies have examined the service. Chen et al. (2015) found that only 141 patients had received the home-based end-of-life service in 2014 [15]. In another local study, the figure was only 21 patients [16]. The number of such patients was much smaller than the number of cancer deaths in Shanghai in the same year, which was 38,000 in 2014 [17]. How home-based end-of-life care is provided and why so few patients take part need to be explored. Therefore, the following research questions were proposed in this study: (i) How is home-based end-of-life care delivered in Shanghai? (ii) What are the factors associated with the delivery of the care?

## Methods

### Aims

The objectives of the study were: (i) to explore how home-based end-of-life care is delivered in community health service centers in Shanghai, and (ii) to examine the difficulties during the delivery of such care.

### Design

This was a qualitative study in which data were collected from interviews and analyzed through qualitative content analysis.

### Informants

Two sets of criteria were developed for recruiting informants: one to screen eligible community health service centers and the other to identify eligible informants. A community health service center was approached if: (a) it was located in Shanghai; and (b) it provided end-of-life care in the community. The end-of-life care could be home-based end-of-life care, inpatient hospice care, or both.

Eligible informants were: (i) those working in a community health service center that had joined the study; (ii) frontline health care providers of home-based end-of-life care, or those in charge of the end-of-life care in the community health service center. Health care providers who did not have any experience in caring for patients at the end-of-life stage or any experience in managing the service were excluded from the study.

The informants were recruited in two steps. First, purposive sampling and snowball sampling were adopted to approach possibly eligible community health service centers. Seventeen such centers located in 14 districts were approached, and 12 (70.6%) agreed to participate. Second, eligible health care providers in the study centers were recruited with the help of the staff of the centers. Those who agreed to participate were given an introduction to the study by telephone or via email several days before the interviews were conducted. They were free to contact the researcher (X.B.L.) if they had any questions about the study. The date of the interview was confirmed after the health care providers fully understood the study and agreed to participate. A total of 19 informants from the 12 centers participated in the study.

### Data collection

Semi-structured interviews were conducted by X.B.L. between August 2018 and February 2019. The interviewer was an academic staff member with a doctoral degree who had 5 years of experience in conducting interviewing and qualitative studies. The informants were interviewed individually in a quiet room in the community health service centers, except for three interviews conducted with two informants. Non-participants were not present during the interviews. A total of 16 interviews were conducted. Fifteen interviews were audio-recorded, and one was not. Field notes were made during this interview, and the researcher wrote down as much of the content as possible immediately after the interview. The interviews ranged in length from 15 to 60 min, with an average length of 35.27 min (*SD*: 13.69). Each informant was interviewed once. Follow-up telephone calls for clarification were made to a few informants. The interview guide is given in Table 1. Data collection ceased when no further centers could be approached within the researchers’ capability.

**Table 1:** Interview guide

Interview questions	
1. What are the criteria for patients to receive the home-based end-of-life care?	
2. Where did the patients come from?	
3. How was the care delivered after the patient was enrolled in the service? Who delivered the care?	
4. What were the care components of the service?	
5. What were the outcomes to the patients under the service?	
6. Please share with me the difficulties and challenges you met when providing the care.	

### Ethical considerations

Ethical approval was obtained from the ethics committee of the university before conducting the study (Reference number: IRB#2019-2-10). The centers and the informants were given assurances that their participation was voluntary and that confidentiality would be maintained. Eighteen informants signed the consent form before the interviews were conducted. One informant gave oral consent and was interviewed without being recorded. The informant stated that she would feel more comfortable to share her experience without recording. The interviewer consulted the ethics committee of the university afterwards whether this health care provider could be an informant of the study and the field note of this interview could be used for analysis. The ethics committee approved.

### Data analyses

All 16 interviews were included for analysis. The interviews were transcribed verbatim. The transcripts were then imported into NVivo software [18] and analyzed using qualitative content analysis [19]. The following steps were taken in the analysis: (a) thoroughly reading the transcripts; (b) dividing the interviews into *content areas* according to the interview questions; (c) extracting and condensing *meaning units*; (d) abstracting and labeling a *code*; (e) sorting the codes into *sub-categories/categories*; and (f) creating *sub-themes/themes* [19]. The data were coded by the first author and reviewed by the other three authors. The codes were then discussed and the first author coded the data a second time, and all the authors reviewed and discussed the codes again, this process being repeated till the final codes and themes were confirmed. The results were discussed by all four female authors. Two authors held a doctoral degree and one a master’s degree, and one was a postgraduate student.

### Rigor

The credibility, transferability, dependability, and conformability of a qualitative study contribute to its rigor [20]. Several methods were used to enhance the rigor of the study. First, the research team tried to find at least one community health service center in each district of the city so as to obtain a comprehensive view of home-based end-of-life care in Shanghai. Second, the doctors, nurses, and team leaders involved in the home-based end-of-life care program in the various centers were interviewed to review the service from different perspectives. Third, all members of the research team were involved in data analysis. Fourth, quotations from the informants were included in the manuscript to improve the transferability and credibility of the study. In addition, the researcher (X.B.L.) kept a personal journal in which thoughts and observations about the study were recorded during data collection and analysis.

## Results

Basic information about the home-based end-of-life care programs of the 12 community health service centers is given in Table 2. In most of the centers, fewer than 10 patients were being given home-based end-of-life care. Among the 19 informants, 7 were physicians, 5 were nurses, and 7 were the leaders of the end-of-life care team in their center. The demographic information of the informants is given in Table 3. Four themes emerged from the interviews: (i) Patients under the home-based end-of-life care; (ii) Service structure; (iii) Service process; (iv) Difficulties in delivering care. The coding tree appears in the Additional file 1: Appendix.

**Table 2:** Basic information about the home-based end-of-life care service

Center ID	Center location	Population in the area in which the center is located	Whether the service was provided in 2017	Number of patients who received the care in 2017	Whether the service was being provided during the study period	Number of patients receiving the care during the study period
C1	Urban area	76,000	Yes	2	Yes	1
C2	Urban area	100,000	Yes	5	Yes	0
C3	Urban area	91,900	Yes	8	Yes	0
C4	Suburb	94,000	Yes	8	Yes	2
C5	Rural area	172,000	No	0	No	0
C6	Rural area	194,000	Yes	20	Yes	5
C7	Rural area	54,000	Yes	1	No	0
C8	Urban area	129,000	Yes	4	Yes	4
C9	Suburb	300,000	Yes	2	No	0
C10	Suburb	210,000	Yes	2	No	0
C11	Rural area	53,000	Yes	3	Yes	1
C12	Urban area	25,000	Yes	2	No	0

**Table 3:** Demographic information of the informants

Item	Mean ± SD	(Min, Max)
Age (year)	41.72 ± 6.84	(27, 56)
No. of years working since graduation (year)	20.22 ± 7.77	(5, 36)
	Median	(Min, Max)
No. of years working in this department (year)	6	(0.1, 33)
	n	%
Gender		
Female	14	73.7
Male	5	26.3
Job role		
Team leader	7	36.8
Physician	7	36.8
Nurse	5	26.4
Educational background		
High school diploma	2	10.5
Bachelor’s degree	15	78.9
Master’s degree	1	5.3
Missing	1	5.3
End-of-life care training		
Yes	16	84.2
No	2	10.5
Missing	1	5.3

### Theme I: patients under the home-based end-of-life care

#### Patient criteria for the service

Several criteria were established to screen eligible patients for the home-based end-of-life service in the study centers. In general, the criteria were broad and flexibly applied. The prerequisite for receiving the service was that the patient be diagnosed with advanced-stage cancer. Another two key indicators were the Karnofsky Performance Score (KPS) and the life expectancy of the patient. The KPS criterion was defined in one community health service center as lower than 40, and in another center as higher than 70. The life expectancy was set to within 1 year. A life expectancy of 3 months was the most common criterion in the study centers.

The condition of the patient was another important aspect of eligibility to receive the service. The patient had to be in a stable condition and conscious, and to have symptoms and problems that could be managed at home.

“We assess the patient’s needs. If his or her problem can be solved at home, I will suggest home care. If not, I will suggest inpatient care.” (Manager No. 2)

Besides the above common criteria, different centers had different criteria for patient eligibility, including being a registered resident of the city, living in the community where the health care center is located, being willing to receive home care, and being open to receiving end-of-life care. In most centers, the last criterion applies to the patient and his/her family, as some patients and families will refuse to receive the care when they know that the service is “end-of-life care”. In only one center was the patient’s awareness of his or her true condition clearly specified to be a criterion for the service.

“The patient must know his or her condition, at least the diagnosis. It is very difficult to deliver the care if the patient does not know.” (Manager No. 2)

It was common for the patients not to know their real condition when they began to receive the home-based end-of-life service.

#### The source of the patients

Most of the patients came to receive the service through one of two routes. One was through the identification of eligible patients by monitoring those with cancer, since the physicians would conduct annual monitoring of the progress of the disease in such patients in the community; the other was to identify such patients at the hospice outpatient clinics of the community health service centers.

“Because we have a hospice ward in the center, some people come to our clinic asking if a family member can be admitted to the hospice ward. We will evaluate the patient first. If the patient is suitable for home care, we will recommend home-based end-of-life care first.” (Physician No. 4)

The families learned about the community end-of-life service from public media and promotional efforts in the community. Some heard about it from others. In one center, the informant mentioned that there was collaboration between a secondary hospital (equivalent to a sub-acute care hospital) and the center.

“They knew about the service from previous patients and their relatives through word of mouth.” (Manager No. 3)

#### The management of the patients

According to the informants, the patients who were receiving the service were managed in two ways. In some centers, the patients were admitted as inpatients, and were called “patients hospitalized at home.” Such patients were managed in the same way as those hospitalized in hospitals. In other centers, the patients were administered in another way, with the service being provided for free. Each method has its own advantages and disadvantages.

“It could help the patient financially if the patient was administered as ‘a patient hospitalized at home’. Home visit fees could be covered by medical insurance. Otherwise, the patient would have to pay the fee by himself/herself.” (Physician No. 2)

“The patients could not go to other hospitals if they were treated as ‘patients hospitalized at home’. This would bring inconvenience to the patients. Hence, we treated the patients in another way, as a free service.” (Manager No. 1)

#### The outcomes of the patients

In most study centers, a proportion of the patients who received the service were admitted to the hospice wards in the community health service centers when their condition deteriorated. The family members believed that the patients could be better cared for in the hospice wards because of the presence of health care providers, more drug choices, and better conditions. They could not handle the symptoms of the patients at home when the symptoms became worse.

“Most families thought staying in a ward is better than staying at home.” (Nurse No. 3)

In some centers, the informants also suggested to the family members that they transfer the patients to the hospice wards for the same reason.

“In the ward, we can resolve more problems. For example, we can control pain intravenously, but we could only give oral drugs for pain if the patients stayed at home.” (Physician No.1)

Another common reason was that many families did not want the patients to die at home.

Besides the hospice wards of the community health service centers, some patients were sent to hospitals by their families. Only in one rural center did the patients die at home because there was no hospice ward in the center.

### Theme II: service structure

#### Service team

In all the study centers, both physicians and nurses were involved in delivering the home-based end-of-life care as the main hospice care providers. In most centers, staff with other backgrounds, such as social workers, volunteers, or psychologists, were seldom involved in home-based end-of-life care. According to the informants, not all the health care providers who delivered the home-based end-of-life care held a certificate in end-of-life care in the study centers.

“In our center, we have a total of 26 physicians providing home-based end-of-life care, but only 6 physicians received the training and have the certificate.” (Physician No.6)

Basically, multi-disciplinary teams were lacking. Although some centers had social workers and volunteers, they were mainly involved in inpatient care. Only in one center would a social worker would follow a home-care case if necessary. Most centers also had no psychologists. The informants did not know if any other services or forms of support were available in the community for patients living at home.

“We have social workers in our center. But they are only involved in the care of patients in the ward.” (Nurse No. 3)

#### Forms of service

Home-based end-of-life care was delivered through multiple approaches, including home visits, telephone follow-ups, and patient-initiated telephone services. Home visits were the most common way of delivering the care. Usually, one doctor and one nurse visited the patient together in the first session, and one or the other would make subsequent home visits. In rural communities, regular telephone calls were the main method for following up on the patients. In all of the study centers, the patients and their families could call the informants whenever they needed to. In addition, one center distributed free pain drugs as a service for advanced cancer patients living at home.

“The physician would ask about the patient’s condition when his/her caregiver came to the outpatient clinic to pick up the free pain drugs.” (Physician No. 7)

In most centers, the service was delivered regularly, ranging from twice per week to once every two weeks. In the other centers, the service was delivered depending on the patient’s condition and needs.

### Theme III: service process

#### Service duration

According to the informants, the patients received the service for a period of 2 weeks to 3 months. Only in one center without a hospice ward did the service last until the death of the patient. The duration of each home visit ranged from 15 min to 60 min in different centers.

“In general, we spent 30 minutes on each home visit. It would be longer on the first home visit, one hour maybe.” (Physician No. 5)

#### Care components

During the first home visit, all of the informants mentioned going through the following procedures, including the recording of their health history, a physical examination, an assessment of the needs of the patient and the family, and the signing of the documents required for receiving end-of-life care.

“We will bring several documents to the patient’s home, such as a consent form for receiving end-of-life care, the final treatment plan (i.e., whether or not the patient should be resuscitated), etc.” (Physician No. 1)

During follow-up sessions, the service consisted of multiple components; including monitoring the condition of the patient, managing the patient’s symptoms, giving daily care instructions, performing nursing procedures, and giving psychological support, but most of the care focused on managing the physical discomfort of the patients and monitoring them.

Monitoring changes in the condition of the patient was an important component of the care. At each visit or call, the informant assessed the patient’s general condition and changes in the cancer and other co-morbidities. Drugs to treat the symptoms were prescribed accordingly. Daily care instructions were also given to the patient and the family caregivers.

“We mainly give them instructions on daily care, for example, how to prevent falls at home, how to prevent pressure ulcers, how to change the position of the patient, etc.” (Nurse No. 1)

Besides providing daily care instructions, the nurses also performed some nursing procedures for the patients, such as dressing wounds, caring for the catheter, drawing blood for tests, and giving intramuscular injections. Some centers prohibited intravenous infusions in home settings, while others permitted one-bottle intravenous infusions. When the home-based end-of-life service was mainly provided by physicians, nurses only visited the patient when necessary, and then mainly to provide functional instead of holistic care.

“The doctor visits the patient first and refers the patient to me if the patient needs a nursing intervention. I do not conduct a holistic assessment of the patient. If the patient needs an intravenous infusion, I will perform it.” (Nurse No. 2)

When asked about psychological support to patients and their families, the informants most commonly mentioned chatting about daily matters with the patients as a way of relaxation.

“I would chat with the patient about daily matters. For instance, I would ask the patient, ‘How is your grandson these days?’ or ‘Your friend visited you today, didn’t he?’ I think this is a more localized way of providing psychological care.” (Manager No. 3)

A few informants also tried to do more in the psychological domain.

“I had a patient with very late stage pancreatic cancer. Every time I visited her, I told her that her condition was better than I expected. These patients needed confidence.” (Nurse No. 3)

There were some types of activities in which they were seldom involved. Surprisingly, talking about death seemed to be beyond the scope of their practice.

“The families and the patients never proposed the topic of death and life to us.” (Nurse No. 4)

Making wills and arranging funerals were two issues in which the informants were seldom involved.

“We have traditions in our rural area. Once a person dies, a grand old man in the village will organize the entire funeral. All of the people in the village will help the family. It is not appropriate for us to become involved.” (Nurse No. 5)

### Theme IV: difficulties in delivering care

#### Unable to carry out the service

The greatest difficulty that the informants encountered was their inability to deliver home-based end-of-life service. One important reason for this was a wide gap between what the service could provide and what the patients and their families needed. The service was established without clear service aims, a sound plan for supplying the service, or well-trained staff.

“The service was established because the presence of the service is a performance indictor for the community health service centers. But we don’t know what exactly we could do for the patients in the service.” (Manager No. 1)

Because of limited supplies, the informants could only provide limited interventions to deal with patients' discomfort. Poor drug supplies were a prominent obstacle.

“We could not prescribe pain drugs for these patients in our center.” (Managers Nos. 5 and 8, Physicians Nos. 1 and 4)

Meanwhile, the informants thought that providing home-based end-of-life care was beyond their ability.

“The general practitioners in our center are good at managing the common chronic illnesses of the elderly. But they lack experience in caring for patients at the end-of-life stage.” (Manager No. 6)

The second reason for not carrying out the service was a lack of patients. Under the perception that the quality of care in community health service centers is poor, people preferred to seek medical care in large hospitals. Even those who went to community health service centers preferred inpatient care to home care. Families believed that patients would receive better care in a ward, and that they would be spared the burden of providing care. They were also concerned with the lack of home-care patients.

“The family members did not want their neighbors to know that there was a patient at home. They were worried about blame from the neighbors. ‘Why not send the patient to the hospital?’ ‘Why let the patient die at home?’ – something like that.” (Manager No. 7)

Sometimes, although patients may have a life expectancy that would qualify them to receive end-of-life care, for example, patients with one-year life expectancy, they might not be in much discomfort, in which case the patient might not consider the service necessary.

The third reason for being unable to carry out home-based end-of-life care was that the staff lacked the motivation to provide such a service. Delivering such care without reasonable rewards was a key factor in killing their motivation.

“Theoretically, the home-base end-of-life care was sponsored by specific funding from the government. However, from the very beginning, we never got the money. We did the job without any rewards. That’s why we stopped the service.” (Physician No. 4)

Meanwhile, a heavy workload further reduced their willingness to deliver the care.

#### Powerlessness when facing psycho-spiritual problems

Although patients at the end-of-life stage have great psychological and spiritual needs, some informants, whose training in end-of-life care was based on content adopted from overseas, felt that what they had learned was of little use when dealing with local Chinese cancer patients.

“I feel very powerless in terms of the psychological aspect. What we were trained in is totally unsuitable for our community.” (Nurse No. 5)

Being unable to tell the patient the truth about his/her condition was the key reason for this feeling. It is common for the home-based end-of-life service to begin without the patient knowing the truth, as requested by the family. It was impossible to initiate any discussion with the patient related to end-of-life issues when the patient was ignorant of his/her real condition.

“Usually the patients did not realize their real condition when receiving the home-based end-of-life care, so our service focused on the physical problems …. In that case, spiritual care was difficult to deliver in home settings.” (Manager No. 7)

A lack of willingness to talk about life and death was another obstacle faced by the informants. Both the patients and their families preferred to “hide behind the door” and avoid discussing the topic openly, even though they both knew the truth. In addition, regular home visits were not a good way to resolve psycho-spiritual problems, according to the informants. The health care providers usually could only spend less than one hour with the patients and their families every 1–2 weeks. They did not think they could establish the strong rapport that would be needed to discuss the subjects of life and death in such a limited time.

#### Technique-related difficulties in delivering care

Besides the main two difficulties mentioned above, the informants mentioned a few practical difficulties, including performing urinary catheterization and venopuncture, the inability to closely monitor the effects of drugs, and finding a suitable time to make home visits.

## Discussion

The aim of home-based end-of-life care is to meet the needs and wishes of the patients at the end-of-life stage and their families. It seems that fewer patients in mainland China choose to receive home care than patients overseas. A recent study found that 43.2% of elderly people in Shanghai preferred to be cared for at home at the end-of-life stage [6]. Another study reported that 58.2% of the elderly people in a nursing home in Wuhan wanted to stay in their current nursing home until death [21]. In a population-based survey conducted in Hong Kong, only 31.2% of the respondents preferred to die at home [7]. These studies indicate that home-based care may be not the most popular choice at the end-of-life stage in mainland China.

The realization that one could stay at home at the end-of-life stage is influenced by multiple factors. In mainland China, health-care-seeking behavior plays a very important role in deciding where to receive care. According to a national report in 2017, there were 30,000 hospitals (3.1%) and 934,000 primary health care institutions (96.9%) in mainland China [22]. In the same year, there were 280 million visits and admissions to hospitals (43.8%), and 360 million visits and admissions to primary health care institutions (56.2%) [23]. Chinese people prefer “large” hospitals, for example, tertiary hospitals in cities, to primary health care institutions. This preference remains influential when the patient is at the end-of-life stage. A recent study found that most patients in Shanghai went to “large” hospitals at the end-of-life stage [24]. The preference of patients and their families for hospitals over home care has contributed the limited patient resources allocated to home-based end-of-life care.

Family caregivers also play a key role in deciding the place of care. Caring for the patient at home is a great burden on family caregivers. In a population-based survey, family caregivers reported spending a median of 69.5 h per week on care-giving [25]. This study reported that the health care providers could only visit the patient once a week or once every two weeks. Thus, the family caregivers shouldered the lion’s share of the care responsibility. In addition, family caregivers may not receive adequate support since there seems to be limited extra support for patients staying at home and their families [26]. Both time and the caring activities themselves pose challenges to family caregivers. A recent review stated that family caregivers often felt unprepared to assist the patient in his/her activities of daily living, in managing the patient’s medications, and in dealing with other practical challenges [27]. The financial burdens of caring for the patient and the changes to their own role during this period were also important practical challenges faced by family caregivers [27, 28]. The condition of the patient is another source of stress for family caregivers. They felt very distressed when the patient’s physical condition was poor [28]. This was a common reason for family caregivers to give up on home-base end-of-life care [25, 29]. Affected by multiple issues and the huge care burden associated with caring activities, family caregivers may finally send the patient to a hospital.

One interesting finding of this study is that the fear of being blamed by neighbors was an obstacle to choosing home-based care. In the view of some traditional Chinese people, letting the patient stay at home is not filial behavior, while sending the patient to a hospital to be rescued by all possible means is a way of showing one’s filial duty. This study also found that such Chinese traditions as hiding the truth from the patient and avoiding talking about death negatively affected the quality of end-of-life care. These behaviors violate the purpose of modern end-of-life care [30]. It is time for people in Chinese society to reconsider the best way of fulfilling their filial duty and of taking care of the patient at the end-of-life stage [31, 32]. Death education is definitely needed to change the minds of Chinese people.

However, the development of death education in mainland China lags far behind that of some other countries. Currently death education is mainly intended for health care providers, and is offered as an elective course in medical and nursing schools [33]. There are few death education programs available in mainland China for patients, their caregivers, and the public [34, 35]. Existing programs are usually adapted from overseas programs, and lack a basis in the local culture [36]. With the rapid development of hospice care, death education for the public has been drawing increasing attention. Health care professionals realize that helping the public develop a proper attitude towards death is essential to developing and expanding end-of-life services in mainland China. This year, during the annual session of National People’s Congress, an oncologist delegate proposed the launching of a death education program for the public [37]. In the future, developing localized programs on life and death education for Chinese people will be an important research direction.

Besides a failure to understand the attitudes and preferences of patients and their families, existing home-based end-of-life care services have been hindered by several factors associated with the health care system. Painlessness and comfort are basic and important needs of patients at the end-of-life stage. However, the drug supply for patients who stay at home is limited. This poses a major obstacle to promoting the service. Meanwhile, this study found that health care providers are not motivated to offer the service because of inadequate financial support and disappointing rewards for doing so. Financial support is critical to maintaining a care program. Some home-based palliative care programs in the United States have also encountered similar challenges [38]. Despite the good intentions behind the call to establish home-based end-of-life care, the government should also devise supportive policies and provide financial support to facilitate the delivery of such care and meet the needs of the patients and their families [39, 40].

Psycho-spiritual wellbeing is extremely important to patients at the end-of-life stage [41–43]. However, the lack of training on psychological and spiritual care makes it difficult for health care providers to practice in clinical settings [44]. Furthermore, it is impossible for health care providers to deepen their care in the psycho-spiritual domain if the patient is not aware of his or her true condition. The traditional Chinese attitudes towards death add further difficulties. Thus far, the ways of providing psycho-spiritual care have been adopted from overseas. Although some care methods or care models have been borrowed from Asian areas with a similar cultural background, the informants found that they were still not suitable for the local patients in this study, and it might be even more difficult to introduce end-of-life care to remote areas of mainland China. The World Health Organization and the World Palliative Care Alliance have suggested that localized end-of-life care be provided to patients [45]. The prerequisite for developing localized care is to understand the recipients of the care. The psychological and spiritual domains of patients at the end-of-life stage in mainland China have not received sufficient attention. Health care professionals need to know what kinds of psycho-spiritual care such patients prefer and who they would prefer to deliver it before localized psycho-spiritual care can be provided to patients at the end-of-life stage.

### Limitations

In Shanghai, there are more than 50 community health service centers providing home-based end-of-life care, yet only 12 eligible centers participated in this study. Some centers refused our invitation. This phenomenon suggests that the development of local home-based end-of-life service might be less optimistic, as well as affecting the data saturation of this study. However, most contents of the interviews were repeatedly mentioned by the informants from different centers during the interviews.

Meanwhile, there are some possible limitations associated with data collection and analysis. First, one interview was not recorded. Although the interviewer took notes during the interview and wrote down as much of the interview content as possible after the interview [46], some information might have been missed, which could affect the richness of the data. Second, the way the authors coded the data may also pose limitations. In this study, the data were coded by the first author and reviewed by the other three authors. Although this is a feasible way to analyze the data [47], the codes generated in the analysis might not be as rich as those generated by multiple coders [47]. In addition, this may impair the credibility of the study, which would be increased through triangulating analyses [46].

The findings of this study shed light on the development of and challenges to end-of-life care in mainland China. However, the fact that it was conducted in a single city is one of the limitations of this study. Differences between sub-cultures in different areas of mainland China should not be ignored. Specific conditions in local areas should be considered when generalizing the findings of this study. In this study, only health care professionals were interviewed about the service. It would have been better to review the service from the perspectives of both health care professionals and patients and their families. In the future, the perceptions of patients and their families on home-based end-of-life care should also be explored.

## Conclusions

A home-based end-of-life service has been offered in Shanghai since 2012 with government support. Each year, only a small number of cancer patients at the end-of-life stage have received this care, despite the broad criteria for patients. The focus of such care is mainly on addressing the physiological problems of the patients instead of on the provision of holistic care. It is difficult to deliver psycho-spiritual care. In the face of two major difficulties (i.e., being unable to carry out the service and a lack of patients), the service seems to have been largely halted. Several issues should be addressed before the service can be further developed, including fully understanding the needs and preferences of local patients and their families, securing stronger financial support and a better supply of drugs, delivering better training for staff, and ensuring greater rewards for individuals and institutions providing the service.

## Supplementary information

**Additional file 1: Appendix.** Illustration of the coding process

## Data Availability

The datasets used and/or analyzed during the current study are available from the corresponding author on reasonable request.

## Abbreviations

KPS: Karnofsky Performance Score
SD: Standard deviation
