# Supplementary material for: An examination of home-based end-of-life care for cancer patients: a qualitative study
Source: BMC Palliat Care. 2019 Dec 16;18:115. doi: 10.1186/s12904-019-0501-y (PMC6915891; doi:10.1186/s12904-019-0501-y)
Supplement: Supplementary file 1 — Additional file 1: Appendix. Illustration of the coding process [file 12904_2019_501_MOESM1_ESM.docx]

**Appendix Illustration of the coding process**

| **Theme** | **Category** | **Sub-category** | **Code** |
| --- | --- | --- | --- |
| **Patients under the** | Patient criteria for the | Prerequisite | Patients with advanced cancer |
| **home-based** | service | Key criteria | KPS score varied |
| **end-of-life** |  |  | Life expectancy within 1 year |
| **care service** |  | Patients' conditions | Being stable |
|  |  |  | Being conscious |
|  |  |  | Problems could be handled at home |
|  |  | Miscellaneous criteria | Being a registered resident of the city |
|  |  |  | Living in the community in which the service could be covered |
|  |  |  | Being open to receiving end-of-life care |
|  |  |  | One center: The patient's awareness of life expectancy/prognosis |
|  |  |  | Being willing to receive home care |
|  | The source of the patients | From cancer surveillance | Identified from cancer monitoring |
|  |  | From the hospice outpatient clinic in the center | Identified from the hospice outpatient clinic in the community health care center |
|  |  | How the patient came to the hospice | Introduced by others |
|  |  | outpatient clinic | Public media & community promotion |
|  | The management of the patients | | Being administered as inpatients |
|  |  |  | Being administered in another way |
|  | The outcomes of the patients | Admitted into the hospice ward | Admitted into the hospice ward of the community health care center |
|  |  | Went to a hospital | To the emergency room |
|  |  |  | Admitted to an inpatient ward |
|  |  | Died at home | At home |

**Appendix Illustration of the coding process (Cont’d)**

| **Theme** | **Category** | **Sub-category** | **Code** |
| --- | --- | --- | --- |
| **Service structure** | Service team | Mainly physicians and nurses | Staff with a qualification certificate for end-of-life care |
|  |  |  | Mixed staff with and without the qualification certificate of end-of-life care |
|  |  | Multi-disciplinary teams were lacking | Social workers and volunteers were not involved in home-based hospice care |
|  |  |  | Social workers followed certain cases |
|  |  |  | No psychologists |
|  |  |  | Not sure if there were any other services or forms of support available in the community |
|  | Forms of service | Home visits | First both of them, doctor mainly |
|  |  |  | First both of them, nurse mainly |
|  |  | Telephone follow-ups | Home visits & telephone follow-ups |
|  |  | Patient-initiated telephone service | Patient-initiated telephone service |
|  |  | Distribution of free pain drugs | Distribution of free pain drugs |
|  |  | Service frequency | Regular: 2 times per week to once every 2 weeks |
|  |  |  | Flexible and dependent on need |
| **Service process** | Service duration | Total duration varied | 0.5 month to 3 months |
|  |  |  | Until death |
|  |  | Duration of each visit:15–60 minutes | 10–20 minutes |
|  |  |  | Around 30 minutes |
|  |  |  | Around 45 minutes |
|  |  |  | Around 60 minutes |
|  | Care components | Must-dos at the initial home visit | Taking the patient’s health history |
|  |  |  | Performing a physical examination |
|  |  |  | Assessing the needs of the patient and family |
|  |  |  | Signing documents on receiving end-of-life care |

**Appendix Illustration of the coding process (Cont’d)**

| **Theme** | **Category** | **Sub-category** | **Code** |
| --- | --- | --- | --- |
| **Service process** | Care components | Condition monitoring | Assessing the patient’s general condition |
|  |  |  | Assessing the progress of the cancer |
|  |  |  | Assessing the changes in co-morbidities |
|  |  |  | Performing a physical examination |
|  |  | Symptom management | Prescribing drugs |
|  |  |  | Controlling presented symptoms |
|  |  | Daily care instructions | Drug instructions |
|  |  |  | Diet instructions |
|  |  |  | Safety suggestions |
|  |  |  | Skin care instructions |
|  |  | Performing nursing procedures | Wound care |
|  |  |  | Catheter care |
|  |  |  | Drawing blood for tests |
|  |  |  | Intramuscular injections |
|  |  |  | Limited intravenous infusions |
|  |  |  | Procedure-based nursing care if referral needed |
|  |  | Psychological support | Chatting about daily matters |
|  |  |  | Attempting to deepen psychological support |
|  |  | Care seldom involved | Talking about death |
|  |  |  | Making wills |
|  |  |  | Funeral arrangements out of scope |

**Appendix Illustration of the coding process (Cont’d)**

| **Theme** | **Category** | **Sub-category** | **Code** |
| --- | --- | --- | --- |
| **Difficulties in** | Unable to carry out the | Wide gap between service and the | Lack of a clear service aim |
| **delivering care** | service | patients and families | Limited interventions for discomfort |
|  |  |  | Limited competence in providing high-quality care |
|  |  | Lack of patients | Prefer large hospitals |
|  |  |  | Prefer inpatient care |
|  |  |  | Obstacles from families |
|  |  |  | No health care needs |
|  |  | Lack of work motivation in staff | Lack of reasonable payment |
|  |  |  | An overload of work |
|  | Powerlessness when | Unable to tell the patient the truth | Family members did not want the patient to know the truth |
|  | facing psycho-spiritual | about his/her condition | The service was begun without patients knowing the truth |
|  | problems |  | Sometimes lost patients’ trust |
|  |  | Unwillingness to talk about life and | Both patients and families prefer to “hide behind the door” |
|  |  | death | The patient did not believe that he was dying |
|  |  | Psycho-spiritual problems could not be | Regular visits are not a good way to provide psycho-spiritual care |
|  |  | well addressed on regular home visits | Lack of rapport |
|  | Technique-related difficulties in delivering care | | Difficulty in performing venopuncture |
|  |  |  | Difficulty in performing urinary catheterization |
|  |  |  | Unable to closely monitor the effects of the drug |
|  |  |  | Difficulty in finding a suitable time for home visits |
